# DEATH OF A GRANDPARENT IN ADULTHOOD: GENDER, GRIEF, AND THE ROLE OF MIDDLE-GENERATION PARENTS

**DOI:** 10.1093/geroni/igz038.2251

**Published:** 2019-11-08

**Authors:** Jeffrey E Stokes, Kyungmin Kim, Karen L Fingerman

**Affiliations:** 1 University of Massachusetts Boston, Boston, Massachusetts, United States; 2 University of Texas at Austin, Austin, Texas, United States

## Abstract

Although the death of a grandparent in adulthood is often an expected or “on-time” life event, this loss may still result in grief for adult grandchildren. Structural aspects of relationships, including gender of the grandparent, adult grandchild, and/or middle-generation parent, may affect the response such a loss elicits from adult grandchildren. Further, adult grandchildren’s relationship quality and/or coresidence with middle-generation parents may also impact the effect of grandparent loss on adult grandchildren’s grief. Using data from Wave 2 of the Family Exchanges Study (2013), we found that (a) grandsons report less grief than granddaughters, irrespective of grandparents’ or middle-generation parents’ gender; (b) relationship quality with and worry about middle-generation parents matter most for granddaughters and those who lost a maternal grandparent; and (c) worry about middle-generation parents matters most for bereaved grandchildren who coreside with middle-generation parents. Results highlight the intersection of gender and relationship quality in a multigenerational context.

